# Integrating rhythmic auditory stimulation in intelligent rehabilitation technologies for enhanced post-stroke recovery

**DOI:** 10.3389/fbioe.2025.1649011

**Published:** 2025-08-29

**Authors:** Yanyan Zhao, Han Xu, Jianming Fu

**Affiliations:** ^1^ Department of Music, Anhui University of Arts, Hefei, China; ^2^ Auckland Tongji Rehabilitaion Medical Equipment Research Center, Tongji Zhejiang College, Jiaxing, China; ^3^ Rehabilitation Medical Centre, The Second Affiliated Hospital of Jiaxing University, Jiaxing, China

**Keywords:** rhythmic auditory stimulation, music stimulation, intelligent rehabilitation, stroke, motor recovery

## Abstract

Stroke remains the leading cause of adult disability worldwide, resulting in long-term motor and cognitive impairments and imposing substantial socioeconomic burdens. Despite the widespread use of rehabilitation therapies, clinical outcomes remain suboptimal, underscoring the urgent need for more effective interventions to enhance neuroplasticity. This review explores the potential of integrating rhythmic auditory stimulation (RAS)—a music-based neurorehabilitation technique that leverages auditory-motor synchronization—with intelligent rehabilitation technologies such as robotics and virtual reality (VR). While various music-based interventions have shown promise in neurological recovery, this review focuses specifically on RAS due to its precise temporal structure, well-established neurophysiological mechanisms, and strong compatibility with technology-assisted platforms. We systematically examine the clinical evidence supporting RAS, evaluate the strengths and limitations of current intelligent rehabilitation systems, and discuss future directions for creating closed-loop, adaptive therapy paradigms. By combining RAS with robotic and VR-based interventions, we propose a novel framework for enhancing motor and cognitive recovery after stroke. This integrated approach offers new opportunities for personalized, engaging, and scalable neurorehabilitation strategies grounded in neuroscience, engineering, and clinical practice.

## 1 Introduction

Stroke is a highly prevalent cerebrovascular disease in adults and has become the leading cause of disability worldwide due to limited recovery potential. The societal impact of stroke is profound, with over 15 million cases annually worldwide, often leading to long-term disability, reduced workforce participation, and substantial healthcare costs exceeding $100 billion per year. Approximately 80% of stroke survivors experience varying degrees of neurological dysfunction, including motor impairments, speech disorders, and cognitive deficits, which severely affect their daily lives (Delfino et al., 2023). Consequently, stroke rehabilitation therapy is essential for improving patient outcomes. Effective rehabilitation can mitigate these burdens by reducing dependency on caregivers and enabling reintegration into daily life. Stroke rehabilitation is a multidisciplinary process designed to promote the recovery of stroke survivors by addressing physical, cognitive, and psychosocial impairments. This comprehensive approach integrates exercise training, neurotechnology-aided technologies, and cognitive training to enhance neuroplasticity, improve functional outcomes, and enhance quality of life (Hendricks et al., 2002).

Although numerous rehabilitation therapies are currently employed in clinical practice, unsatisfactory treatment outcomes highlight the ongoing challenge of developing effective approaches to enhance neuroplasticity after stroke. From a scientific standpoint, advancing stroke rehabilitation technologies holds transformative potential. Research in this field not only deepens our understanding of neuroplasticity and motor-cognitive recovery mechanisms but also drives innovation in intelligent rehabilitation, including robotics and virtual reality (Zhou et al., 2022). These technologies serve as experimental platforms to test hypotheses about brain repair while offering scalable solutions for personalized medicine. By integrating cutting-edge tools like AI-driven adaptive therapy and rhythmic neuromodulation, rehabilitation science bridges gaps between neuroscience, engineering, and clinical practice. Ultimately, breakthroughs in this domain promise to redefine recovery paradigms, offering hope for millions while alleviating global healthcare disparities.

**FIGURE 1:**
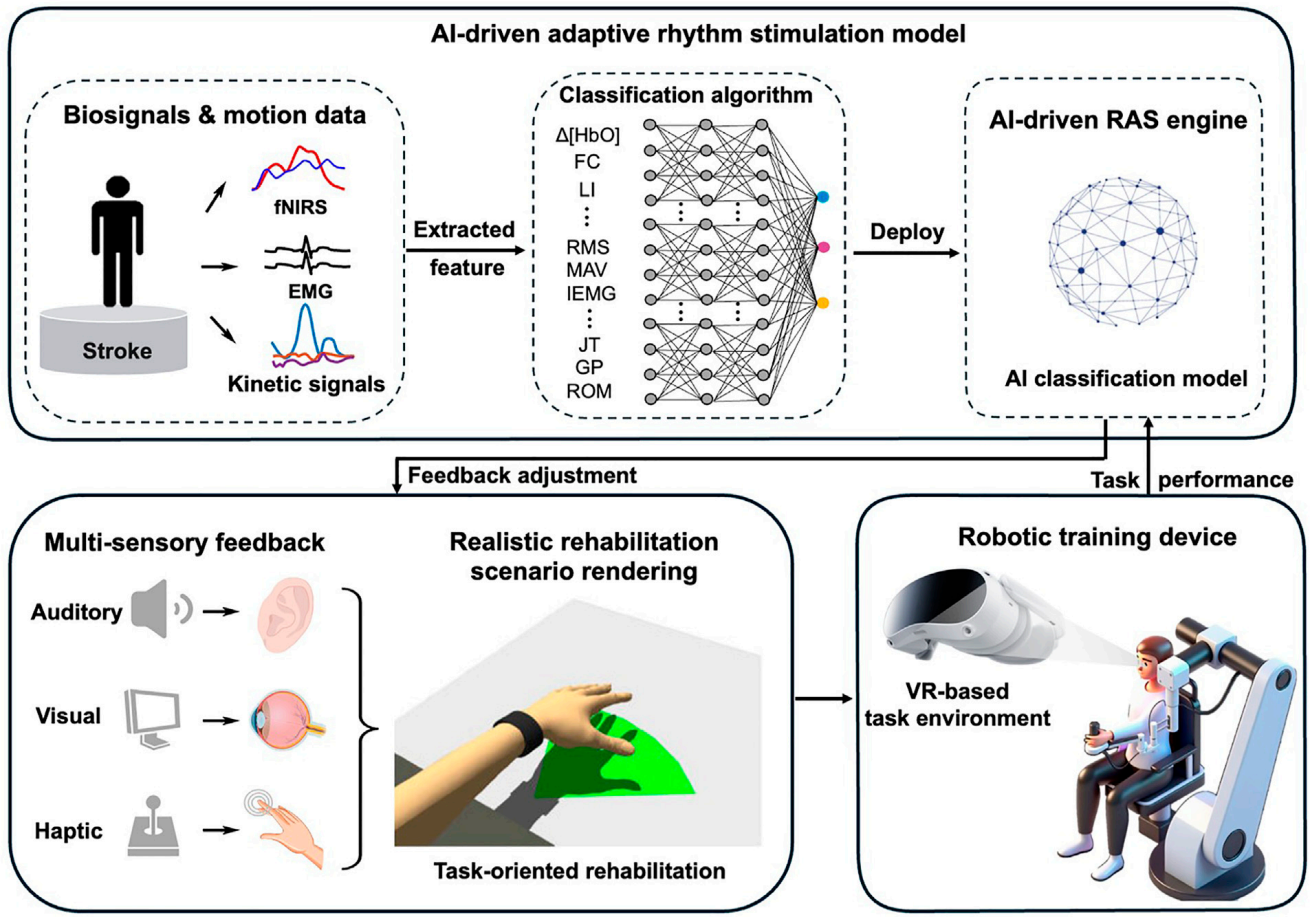
Conceptual model of AI-Driven adaptive rhythmic stimulation in intelligent stroke rehabilitation.

Given the need to enhance the clinical efficacy of current rehabilitation technologies and the established theory that multimodal feedback can amplify neuroplasticity, this study focuses on developing an integrated approach that synergistically combines rhythmic auditory stimulation (RAS) with existing intelligent rehabilitation interventions (Figure 1). While diverse music-based interventions (e.g., music listening, singing) show rehabilitative potential, this review specifically focuses on RAS due to three primary reasons: (1) its precise temporal entrainment capability for motor synchronization; (2) it established neurophysiological mechanisms promoting neuroplasticity; and (3) its technical compatibility with intelligent rehabilitation platforms, including robotics and VR. Other interventions, although promising, currently lack sufficient empirical evidence or standardization for integration with emerging neurorehabilitation technologies.

RAS is an evidence-based neurorehabilitation technique that employs external rhythmic auditory cues—such as metronome beats or musical rhythms—to facilitate motor function recovery. Grounded in the principle of auditory-motor synchronization, RAS has been shown to enhance gait regulation, balance control, and motor coordination. It has been extensively utilized in the rehabilitation of movement disorders resulting from neurological conditions, including stroke, parkinson’s disease, and multiple sclerosis, yielding significant clinical efficacy (Gonzalez-Hoelling et al., 2022). However, the integration of this approach with robotic-assisted training and VR remains insufficiently explored, despite the complementary advantages offered by these technologies: robotic systems deliver precise kinematic feedback, whereas VR promotes user engagement through immersive, task-oriented environments. This study will systematically investigate how RAS can be embedded into smart rehabilitation systems to create a closed-loop, adaptive therapy paradigm. Specifically, we will systematically examine (1) the clinical evidence supporting RAS in motor and cognitive recovery, (2) the efficacy of current intelligent rehabilitation technologies, and (3) what are the promising future directions and strategies for implementation. By integrating rhythmic neuromodulation with intelligent rehabilitation technologies, this research aims to establish a novel, evidence-based framework that maximizes neuroplasticity and functional recovery, ultimately advancing personalized and scalable stroke rehabilitation strategies.

## 2 Rhythmic auditory stimulation for stroke

RAS holds great potential for enhancing neurorehabilitation after stroke. The strongest evidence for the efficacy of RAS interventions has been reported for stroke. Four recent systematic reviews (Table 1) have documented the benefits of RAS as an adjunctive intervention for post-stroke neurological impairments (Table 1).

**TABLE 1:** Systematic reviews on rhythmic auditory stimulation for post-stroke recovery.

Studies	Year	Databases	Samples	Indicators	Conclusion
(Ghai and Ghai, 2019)	2019	Nine databases: Web of science, PEDro, EBSCO, Scopus, MEDLINE, Indian citation index, Cochrane central register of controlled trials, EMBASE, PROQUEST.	38 studies with 968 stroke patients	Gait velocity, stride length, cadence, timed-up and go test	Beneficial effects of training with auditory cueing on gait and postural stability, and strongly recommends the incorporation of rhythmic auditory cueing in gait and postural rehabilitation
(Wang et al., 2022)	2022	Six databases: Medline, Web of Science, Embase, PubMed, Wanfang, China National Knowledge Infrastructure	22 studies were included, 18 studies were included for meta-analysis	Motor function: step length, step cadence, velocity, Fugl–Meyer assessment; balance: overall balance index, Berg Balance Scale	RAS could improve gait parameters, walking function, and balance ability of individuals with stroke
(Scataglini et al., 2023)	2023	Five databases: PubMed, PEDro, Medline, Web of Science, Science Direct	15 studies were considered	Spatiotemporal gait parameters: speed, stride length, cadence, and ROM	The findings confirm the value of RAS as a favorable and effective tool to implement for the rehabilitation of patients with movement disorders
(Gonzalez-Hoelling et al., 2024)	2024	Six databases: PubMed, PEDro, Cochrane Central Register of Controlled Trials, Web of Science, Scopus, CINAHL electronic databases	21 studies with 948 stroke patients	Functional ambulation ability and the use of walking assistive devices. Functional independence, falls and quality of life. such as the Functional Independence Measure (FIM), the Barthel Index, and the modifed Rankin scale	The most consistent fnding was that RAS improves walking and balance parameters in stroke patients in all phases compared to baseline and versus control groups with conventional treatment

Ghai and Ghai (2019) performed a systematic review and meta-analysis to analyze the effects of auditory cueing on gait and postural stability post-stroke. Out of 1,471 records from nine academic databases, 38 studies involving 968 patients were finally included. The results strongly suggest the incorporation of rhythmic auditory cueing-based training in gait and postural rehabilitation, post-stroke. Wang et al. (2022) conducted a systematic review and meta-analysis to summarize the effectiveness of RAS for the treatment of motor function and balance ability in stroke patients. The 22 studies were included in the review, of which 18 studies were included in the meta-analysis due to the availability of data. The results indicated that RAS could improve the gait parameters, walking function, and balance function of individuals with stroke. It is noted that the authors also recommend using a larger sample size and a more rigorous design to obtain strong conclusions about the advantages of RAS in the future. Scataglini et al. (2023) conducted a systematic review following PRISMA 2020 guidelines to evaluate the effect of music-based RAS using wearable devices in neurological rehabilitation. Results from 15 qualified studies showed significant values of spatiotemporal gait parameters of music-based RAS, mainly including speed, stride length, cadence, and Range of Motion (ROM). Gonzalez-Hoelling et al. (2024) recently conducted a systematic review including 21 studies involving 948 stroke survivors. The results reveal considerable heterogeneity among studies investigating the effects of RAS in stroke patients, and the overall evidence regarding its clinical efficacy remains inconclusive. Nonetheless, the best available evidence suggests that functional ambulation in individuals with chronic stroke improves following a RAS intervention, with gains exceeding those achieved through no treatment or conventional therapy.

It is clear that RAS has emerged as a promising adjunctive therapy for stroke rehabilitation, with robust evidence supporting its efficacy in addressing post-stroke neurological impairments. The strongest therapeutic benefits of RAS have been demonstrated in gait rehabilitation, where it enhances motor timing, balance, and interlimb coordination through neural entrainment mechanisms. The underlying neurophysiological mechanisms involve the modulation of corticomotor excitability and the facilitation of sensorimotor integration, particularly in the lesioned hemisphere. RAS leverages the brain’s inherent responsiveness to rhythmic cues, promoting neuroplasticity and functional reorganization in motor networks. Clinical applications extend beyond gait training, with emerging evidence supporting its use for upper-limb motor recovery and cognitive rehabilitation.

Despite these promising outcomes, the optimal parameters for RAS (e.g., tempo, rhythm complexity, and session duration) remain to be standardized. Future research should focus on personalized RAS protocols and their integration with emerging technologies, such as robotics and virtual reality, to maximize therapeutic outcomes in stroke rehabilitation.

## 3 Existing intelligent rehabilitation technologies

Contemporary intelligent rehabilitation technologies for stroke recovery primarily include robotic systems and VR platforms, both of which demonstrate significant clinical potential. Robotic devices deliver precise, repetitive motor training with real-time feedback. These systems—ranging from exoskeletons (e.g., Lokomat for gait training) to end-effector devices (e.g., MIT-Manus for upper limbs)—leverage force feedback and adaptive algorithms to deliver personalized motor retraining (Alashram et al., 2021; Klamroth-Marganska et al., 2014). By providing high-dose, task-specific movements, robots address paresis and spasticity while reducing therapist dependency. Advanced models integrate real-time biosignal monitoring, such as surface electromyography (sEMG) and electroencephalography (EEG), to synchronize assistance with patient intent (López-Larraz et al., 2024), promoting neuroplasticity through Hebbian learning principles. However, limitations persist: many robots focus narrowly on isolated joint movements, lacking ecological validity for daily activities. Recent innovations aim to mitigate these issues—for example, soft exosuits with textile-based actuators improve comfort and portability, while AI-driven controllers dynamically adjust assistance based on performance. Despite progress, robotic therapy alone often fails to engage cognitive-emotional networks critical for holistic recovery, underscoring the need for multimodal integration.

VR-based rehabilitation immerses patients in interactive, simulated environments to restore motor and cognitive functions post-stroke. By combining gamification with real-time motion tracking, VR systems enhance engagement through goal-directed tasks like virtual grocery shopping or obstacle avoidance. These platforms excel in delivering contextual training that bridges clinic-to-home transitions, with studies showing 20–40% greater adherence compared to conventional therapy. Cognitive benefits are particularly notable: dual-task VR paradigms (e.g., stepping while solving math problems) improve executive function by simultaneously activating prefrontal and motor cortices (Fishbein et al., 2019). Nevertheless, challenges remain—current VR solutions often lack haptic feedback, limiting their ability to retrain force modulation. Emerging metaverse applications propose social VR environments for group therapy, yet clinical evidence remains sparse. Crucially, most VR systems overlook rhythmic auditory stimulation, missing an opportunity to leverage music’s proven neuromodulatory effects on motor timing and mood regulation, which could amplify therapeutic outcomes.

While robotic-assisted rehabilitation excels in delivering precise, high-intensity motor training and VR systems enhance engagement through immersive cognitive-motor tasks, both approaches exhibit complementary limitations. Robots often lack ecological validity and cognitive integration, whereas VR frequently misses haptic feedback and rhythmic neuromodulation. A synergistic framework merging robotic precision with VR’s contextual adaptability—augmented by rhythmic auditory stimulation—could potentially address these gaps. By embedding force feedback into VR environments and synchronizing robotic assistance with RAS-enhanced tasks, this integrated paradigm may simultaneously optimize motor recovery, cognitive engagement, and neuroplasticity.

## 4 Future directions

Stroke rehabilitation stands to benefit significantly from the strategic integration of RAS with emerging technologies. RAS has demonstrated unique potential to enhance neuroplasticity by entraining neural oscillations, yet its systematic combination with two key rehabilitation platforms—robot-assisted therapy and metaverse-based training—remains underexplored. This paper highlights two critical research directions to advance the field: (1) optimizing RAS-robot synchronization to amplify motor recovery, and (2) developing RAS-metaverse integration frameworks to boost cognitive-motor outcomes (Figure 1).

### 4.1 RAS-robot synchronization to amplify motor recovery

The first direction focuses on enhancing robot-assisted therapy through RAS integration. By identifying optimal rhythm parameters and developing closed-loop systems that adapt to patient performance, this approach aims to improve corticospinal excitability and adherence. Early studies suggest RAS-robot training could improve stable balance recovery and gait abilities over conventional robotics, potentially redefining standards for movement recovery (Park, 2025). The second direction explores RAS integration with metaverse rehabilitation to address cognitive-motor coupling. This involves designing adaptive soundscapes that respond to user performance in VR tasks and creating synchronized multi-user environments. Preliminary data indicate such integration may increase training duration, offering new possibilities for community-based rehabilitation. Together, these directions represent a transformative step toward personalized, neuroplasticity-optimized stroke recovery (Farsi et al., 2025).

To realize the full potential of these hybrid systems, it is crucial to understand how each modality contributes functionally within the integrated framework. Specifically, examining the complementary roles of RAS, haptics, and immersive media reveals how multi-sensory synergy may drive motor and cognitive recovery more effectively.

RAS complements haptic feedback by aligning temporal auditory cues with proprioceptive inputs. For instance, rhythmic beats can time the initiation of limb movement, while synchronized haptic pulses from an exoskeleton reinforce the correct trajectory and force output. This temporal coherence enhances motor prediction and execution, helping to consolidate sensorimotor pathways through repeated, feedback-rich movement cycles. In virtual reality (VR) settings, auditory cues interact with visual stimuli by reinforcing attention, anticipation, and motion planning. When rhythmic sounds are aligned with visual task events—such as footsteps on a virtual path or object grasping in a game—this cross-modal congruence improves perceptual salience and user engagement, facilitating more naturalistic and effective motor learning.

### 4.2 Developing RAS-metaverse integration frameworks to boost cognitive-motor outcomes

With the development of artificial intelligence technologies such as large models, AI-powered integration of RAS in smart rehabilitation interventions has become feasible. The integration of RAS into intelligent rehabilitation systems presents a transformative opportunity to enhance neurorecovery through personalized, multimodal feedback. By leveraging artificial intelligence, we can develop dynamic systems that not only synchronize auditory cues with rehabilitation tasks but also incorporate emotionally resonant vocal encouragement from loved ones, creating a truly patient-centered therapeutic experience. This approach combines three key technological innovations to optimize engagement and outcomes.

First, AI-driven adaptive rhythm generation enables real-time alignment of auditory cues with movement kinematics. Deep reinforcement learning algorithms analyze motion capture data (e.g., joint angles, velocity) and physiological signals (EMG/EEG) to produce precisely timed rhythmic patterns that reinforce motor learning. For upper-limb reaching tasks, the system generates rhythmic accents coinciding with movement initiation and termination points, leveraging the brain’s sensitivity to temporal predictability to improve movement smoothness (Fan et al., 2022). The auditory-motor synchronization strengthens corticocerebellar loops critical for movement automatization.

Second, emotional voice modulation transforms recorded messages from caregivers into musically structured encouragement. Using generative adversarial networks, the system preserves the speaker’s vocal identity while applying musical parameters (tempo, pitch contour) that maximize motivational impact. When fatigue is detected via facial expression analysis or heart rate variability, the AI shifts to major-key harmonies and brighter timbres—an approach shown to increase training persistence. This personalized auditory feedback taps into the limbic system’s responsiveness to familiar voices and musical emotion.

Third, a multisensory integration platform ensures temporal coherence (<50 ms latency) between rhythmic cues, robotic haptic feedback, and VR visual stimuli. Cross-modal attention mechanisms prioritize the most salient sensory inputs based on individual impairment profiles (e.g., emphasizing auditory cues for patients with proprioceptive deficits). fMRI studies confirm this approach enhances activation in multisensory integration, accelerating functional connectivity restoration (Alwashmi et al., 2024).

### 4.3 Closed-loop RAS-VR-robot integration in practice

In a typical rehabilitation session, a stroke patient engages with an integrated system that combines RAS, robotic assistance, and immersive virtual reality. The patient wears a wearable robotic exosuit that provides lower-limb support while navigating a virtual environment, such as a simulated home or grocery store. Rhythmic auditory cues, embedded within music or metronome patterns, are synchronized with the patient’s intended movements to facilitate step timing and interlimb coordination. The robotic device delivers haptic feedback aligned with the rhythmic cues, reinforcing sensorimotor learning through temporal coherence.

As the patient interacts with goal-oriented tasks in the VR environment—such as obstacle avoidance, reaching for virtual objects, or dual-task activities—the system continuously monitors motor performance using motion analysis and biosignals (e.g., EMG, EEG). An AI-driven control module dynamically adjusts the tempo and complexity of the auditory stimuli and modulates task difficulty to maintain optimal engagement. Emotional vocal cues or musical phrases may be introduced during signs of fatigue to sustain motivation. This closed-loop, multisensory system enables adaptive, high-intensity training tailored to individual neurophysiological profiles, ultimately enhancing neuroplasticity and accelerating functional recovery.

## 5 Discussion and conclusion

Post-stroke neural functional recovery is highly heterogeneous, influenced by factors such as lesion location and extent, time since onset, baseline motor and neural status, and individual neuroplastic potential. This variability often results in inconsistent outcomes when standardized rehabilitation protocols are applied across patients. Addressing this challenge requires tailoring stroke neurorehabilitation strategies to individual needs. Precision medicine aims to ensure that each patient receives the appropriate treatment at the optimal dose and timing (Yan et al., 2025). In this approach, patients are first stratified according to clinical and neurophysiological biomarkers—such as motor evoked potentials, lesion mapping, and functional connectivity profiles—and then assigned intervention protocols, including RAS parameters, that are adapted to their specific characteristics and responsiveness. Moreover, treatment plans should be dynamically adjusted throughout the rehabilitation process based on continuous performance metrics and neurophysiological feedback.

By integrating multimodal assessments, such as fNIRS, EEG, and EMG, with predictive modeling, clinicians can design personalized rehabilitation trajectories that optimize both neural reorganization and functional recovery, thereby reducing inter-patient variability in outcomes. This individualized framework aligns with the emerging paradigm of precision neurorehabilitation and holds substantial promise for enhancing the clinical efficacy of RAS-based interventions across diverse stroke populations (Hakon et al., 2017).

Real-time monitoring of neural and motor states is a critical prerequisite for implementing truly adaptive, patient-specific rehabilitation strategies. fNIRS enables continuous, non-invasive measurement of cortical hemodynamic responses, such as changes in oxygenated hemoglobin concentration within motor-related brain regions (e.g., the primary motor cortex and supplementary motor area), thereby reflecting neural engagement and motor preparation. Simultaneously, EMG records muscle activation timing, amplitude, and co-contraction patterns, providing valuable insights into motor execution quality and coordination (Zhao et al., 2024; Klein et al., 2018).

By integrating these two modalities, multimodal signals can be processed in real time to extract relevant features, including cortical activation magnitude, muscle onset latency, and inter-muscle coherence. Building on these features, an AI-driven RAS engine—employing machine learning methods such as reinforcement learning or Bayesian optimization—can dynamically adapt stimulation parameters. These parameters may include: (i) tempo (beats per minute), fine-tuned to match or slightly challenge the patient’s current motor performance speed; (ii) rhythmic complexity, progressively increased from simple isochronous beats to more complex rhythmic cues as motor control improves; (iii) cue modality and intensity, modulated in terms of timbre, volume, or spatialization to optimize attentional engagement; and (iv) phase alignment, adjusted relative to motor output to reinforce or correct movement timing. The adaptation follows a closed-loop framework in which: (1) fNIRS and EMG data are acquired during task performance; (2) neuro-motor performance indicators (e.g., increased primary motor cortex oxygenation, reduced EMG variability); are computed; (3) RAS parameters are updated via the AI model to maximize these indicators; and (4) the stimulation strategy is iteratively refined across sessions (Fedotchev, 2022; Chandrabhatla et al., 2023). To further optimize this process, multi-objective learning algorithms can be employed to balance gains in neural activation with improvements in functional motor performance, such as gait velocity and step symmetry. Over time, this approach enables the AI engine to learn individualized parameter trajectories that accelerate recovery while minimizing fatigue and cognitive overload.

The integration of RAS with intelligent rehabilitation technologies is supported by three well-established neurophysiological mechanisms: (1) Neural oscillation and rhythmic entrainment; (2) Activation of dopaminergic pathways; (3) Neural activation and plasticity.

### 5.1 Neural oscillation and rhythmic entrainment

**FIGURE 2:**
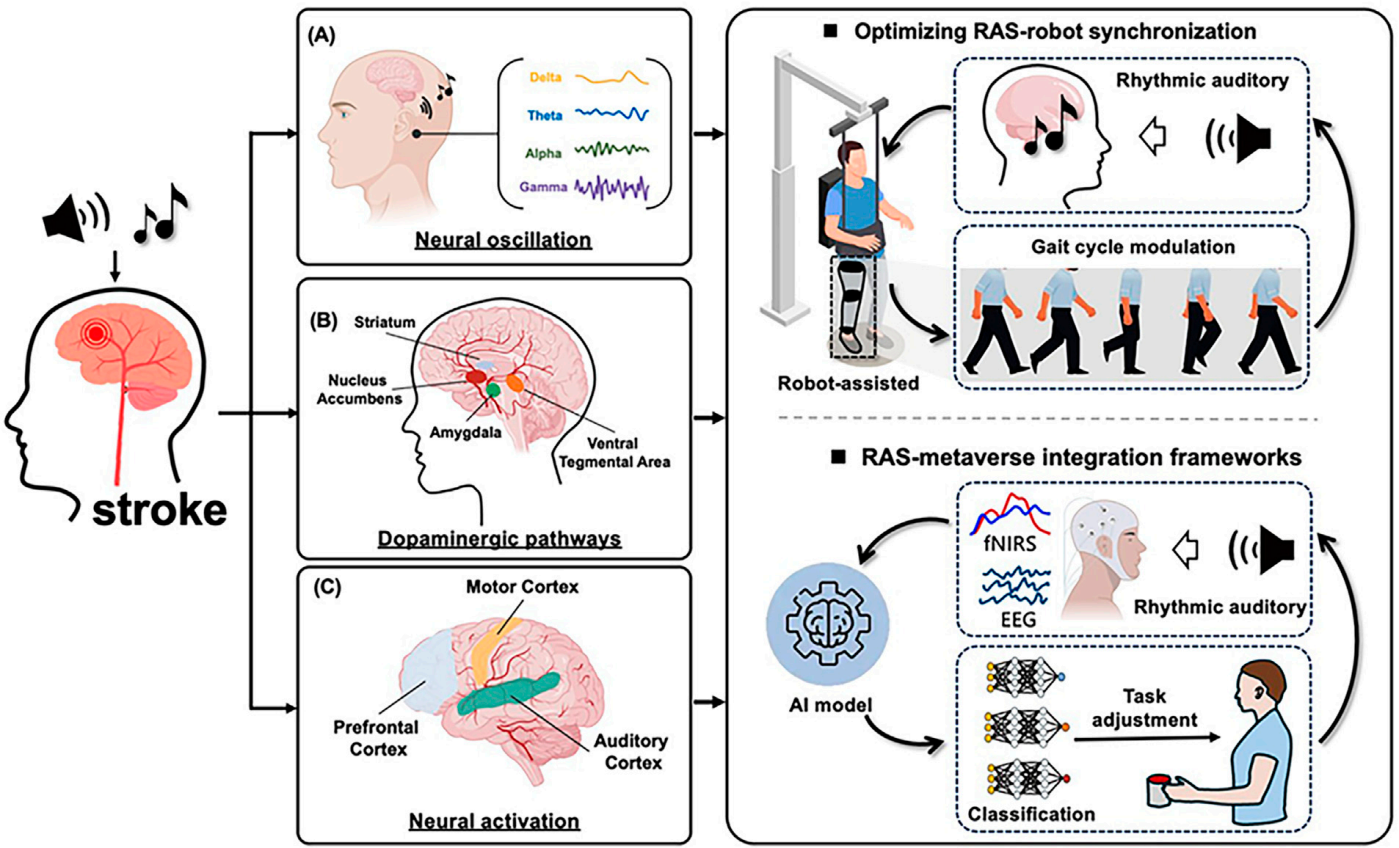
Neural mechanisms and future directions of RAS-Promoted functional recovery in stroke. **(A)** Neural oscillation and rhythmic entrainment. **(B)** Activation of dopaminergic pathways. **(C)** Neural activation and plasticity.

Auditory rhythmic stimulation induces neural entrainment, particularly in the gamma-band, which drives phase-locked oscillations in motor cortical regions (Figure 2A). This modulation stabilizes beta-band oscillations within the basal ganglia–thalamocortical loop, resulting in reduced motor response latency (by approximately 30%–40%). Furthermore, theta–gamma cross-frequency coupling enhances communication between prefrontal and motor cortices, facilitating improved motor planning during complex tasks. Notably, VR-based motor training incorporating rhythmic auditory cues has demonstrated superior motor accuracy, highlighting the synergy between sensorimotor timing and cortical oscillatory dynamics (Leite et al., 2020).

Clinical studies have provided converging evidence for these neural mechanisms. For instance, gait training with rhythmic auditory stimulation in post-stroke patients has been shown to significantly increase beta-band power in the primary motor cortex and supplementary motor area, correlating with improved step symmetry and reduced gait variability (Altenmüller et al., 2009). Similarly, MEG recordings have demonstrated enhanced gamma-band coherence between auditory and motor cortices following RAS-based upper limb training, which was associated with faster reaction times and greater movement smoothness. Moreover, a recent fNIRS–EEG study reported that VR-based motor training incorporating rhythmic auditory cues elicited stronger oxygenated hemoglobin responses in M1 and premotor regions, alongside increased beta desynchronization, both of which predicted greater gains in motor accuracy (Wang et al., 2022). These findings collectively support the hypothesis that rhythmic entrainment promotes motor recovery by enhancing cross-frequency coupling and stabilizing motor-related oscillatory networks.

### 5.2 Activation of dopaminergic pathways

Rhythmic auditory cues, particularly music, activate the mesolimbic dopaminergic pathway, enhancing patient engagement via reward anticipation and emotionally salient feedback (Figure 2B). This activation is associated with elevated extracellular dopamine, which supports cognitive and emotional recovery in individuals with neurological disorders. The inclusion of familiar voices or musical patterns further strengthens intrinsic motivation. Additionally, multisensory feedback loops—combining auditory cues with synchronized haptic or proprioceptive input—intensify the reward experience and consolidate sensorimotor learning (Salimpoor et al., 2011).

Clinical studies have corroborated these mechanisms. Functional neuroimaging in stroke patients undergoing music-based therapy has revealed increased activation in the ventral striatum and nucleus accumbens—key dopaminergic hubs—correlating with higher patient-reported motivation and adherence to training (Särkämö et al., 2014). In a PET study involving rhythmic auditory stimulation during a finger-tapping task, participants exhibited significantly reduced tapping period error and variability (*p* = 0.007 and p = 0.006). Importantly, the presence of RAS resulted in decreased dopamine-binding variability in the left ventral striatum (*p* = 0.013), suggesting that external rhythmic cues increase striatal dopamine release, which is associated with improvements in both motor timing and affective state (Koshimori et al., 2019). Moreover, a randomized controlled trial found that combining rhythmic auditory stimulation with tactile cues significantly enhanced gait velocity and stride length in chronic stroke patients, with concomitant increases in functional connectivity between auditory and reward-related brain regions (Rodriguez-Fornells et al., 2012). These findings support the hypothesis that dopaminergic system activation mediates the motivational and motor benefits of rhythmic auditory cueing, thereby reinforcing long-term rehabilitation gains.

### 5.3 Neural activation and plastic

Modern intelligent rehabilitation devices (e.g., exoskeletons and VR systems) enhance neural plasticity by precisely timing rhythmic stimuli to align with motor intent (Figure 2C). Exoskeletons synchronize torque impulses with RAS to facilitate corticocerebellar integration, while VR platforms leverage the brain’s predictive coding mechanisms to refine motor representations. Evidence from multimodal neuroimaging studies demonstrates that combined RAS and robotic training promotes white matter integrity (e.g., in the acoustic radiation and corticospinal tracts) and functional connectivity within auditory–motor networks, suggesting durable neuroanatomical changes conducive to recovery (Zaatar et al., 2024). Clinical evidence supports these effects. For example, Yang et al. used diffusion tensor imaging (DTI) to examine stroke patients before and after 6 weeks of robot-assisted gait training combined with RAS. Results showed significant increases in fractional anisotropy within the corticospinal tract, correlating with improved gait velocity and balance scores (Yang et al., 2017). In another randomized controlled trial, Park and Chung applied VR-based motor training with synchronized rhythmic cues to chronic stroke survivors. fMRI revealed enhanced connectivity between the supplementary motor area and auditory cortex, accompanied by greater improvements in step symmetry compared with VR training alone (Park and Chung, 2018). These findings suggest that integrating rhythmic cues into intelligent rehabilitation devices promotes not only motor recovery but also measurable structural and functional brain reorganization.

This integrative approach represents a paradigm shift from conventional rehabilitation by actively driving neural reorganization through biologically embedded rhythmic processing. The framework combines neural oscillation, dopaminergic reward mechanisms, and neural plasticity to create closed-loop systems that adapt to real-time neurophysiological biomarkers.

Despite the promise of RAS-integrated intelligent rehabilitation, several key challenges remain that may limit its widespread clinical application. First, latency and synchronization across multiple sensory modalities—such as aligning RAS cues with haptic and visual feedback—present technical difficulties. Even minimal asynchrony can impair sensorimotor integration, reducing training effectiveness. Second, the parameter tuning of rhythmic stimuli (e.g., tempo, beat salience, cue periodicity) is currently guided more by empirical observation than by individualized neurophysiological profiles. Third, inter-subject variability in responsiveness to rhythmic cues complicates the design of universal therapeutic protocols, emphasizing the need for adaptive systems tailored to individual neural and behavioral characteristics.

Addressing these limitations is essential for advancing the field. Future development should prioritize real-time synchronization algorithms, biomarker-driven stimulus optimization, and adaptive closed-loop architectures that respond dynamically to patient-specific neurophysiological feedback (e.g., EEG, EMG). These improvements will enhance the precision and scalability of RAS-based interventions. Ultimately, this integrative approach represents a paradigm shift from conventional rehabilitation by actively driving neural reorganization through biologically embedded rhythmic processing. By combining neural oscillation, dopaminergic reward mechanisms, and neuroplasticity within an intelligent, feedback-sensitive framework, this strategy has the potential to transcend mechanical assistance and harness the brain’s innate temporal processing capabilities. Future research should also aim to validate neuroplastic outcomes through large-scale, rigorously designed clinical trials, solidifying the scientific foundation of this music neuroscience–rehabilitation engineering synthesis.

## Data Availability

The original contributions presented in the study are included in the article/supplementary material, further inquiries can be directed to the corresponding author.
